# Insights into Advanced Neurological Dysfunction Mechanisms Following DBS Surgery in Parkinson’s Patients: Neuroinflammation and Pyroptosis

**DOI:** 10.3390/cimb45050284

**Published:** 2023-05-20

**Authors:** Hao Meng, Jia-Hang Wei, Peng-Zheng Yu, Jia-Xin Ren, Meng-Yao Tang, Jun-Yi Sun, Xiao-Yu Yan, Jing Su

**Affiliations:** Department of Pathophysiology, College of Basic Medical Sciences, Jilin University, 126 Xinmin Street, Changchun 130021, China; menghao@jlu.edu.cn (H.M.); weijh7019@mails.jlu.edu.cn (J.-H.W.); yupz7019@mails.jlu.edu.cn (P.-Z.Y.); renjiaxin97@163.com (J.-X.R.); tangmy2719@mails.jlu.edu.cn (M.-Y.T.); junyisun2000@163.com (J.-Y.S.)

**Keywords:** Parkinson’s disease, DBS surgery, pyroptosis, neuroinflammation, neuroprotective

## Abstract

Parkinson’s disease is a severe neurodegenerative disorder. Currently, deep brain electrical stimulation (DBS) is the first line of surgical treatment. However, serious neurological impairments such as speech disorders, disturbances of consciousness, and depression after surgery limit the efficacy of treatment. In this review, we summarize the recent experimental and clinical studies that have explored the possible causes of neurological deficits after DBS. Furthermore, we tried to identify clues from oxidative stress and pathological changes in patients that could lead to the activation of microglia and astrocytes in DBS surgical injury. Notably, reliable evidence supports the idea that neuroinflammation is caused by microglia and astrocytes, which may contribute to caspase-1 pathway-mediated neuronal pyroptosis. Finally, existing drugs and treatments may partially ameliorate the loss of neurological function in patients following DBS surgery by exerting neuroprotective effects.

## 1. Introduction

Parkinson’s disease is a complicated chronic progressive neurological illness that affects more than six million people worldwide [1]. The incidence of Parkinson’s disease is continuing to increase as the world’s population ages at an accelerating rate, placing a significant financial burden on patients, their families, and national medical insurance organizations. As such, the development of effective therapeutic strategies is critical [2]. Dopaminergic medications, such as Levodopa and Amantadine, are currently the most common drug classes in clinics. However, as the condition worsens, dopaminergic neurons in the substantia nigra and striatum deteriorate, decreasing the capacity of endogenous dopamine to be stored and released and causing the therapeutic benefit of dopaminergic medicines to wane progressively [3]. Surgical treatment is becoming increasingly popular owing to its quick control, effectiveness, and low recurrence [4]. The primary surgical treatments for Parkinson’s disease include radiofrequency ablation [5], and deep brain stimulation surgery (DBS) of the subthalamic nucleus (STN) and globus pallidus internal (GTi) [6], in addition to new therapies such as gene therapy, gamma knife, MRgLITT [7], and MRgFUS [8]. One guiding concept of such surgery is to destroy a target neuron in a certain direction to halt aberrant EEG activity [9]. Currently, only a few patients with late-onset refractory Parkinson’s disease and contraindications for DBS are regularly treated with this radiofrequency ablation technique in neurosurgery clinics [10]. The other method is based on the principle of DBS surgery, which involves implanting electrodes into the deep brain nuclei to generate continuous electrical pulse stimulation through an implantable pulse generator (IPG) to improve patients’ fine movement [11]. This technique is effective for primary PD; however, its negative effects have become increasingly apparent. Only 43% of the patients receiving STN-DBS who participated in the survey were happy with the outcomes. Depression, suicide, apathy, and postoperative disruption of consciousness were the most common adverse effects [12]. In a comparative study of STN-DBS and GPI-DBS, STN-DBS was associated with a higher incidence of postoperative cognitive impairment than GPI-DBS [13]. Further studies suggest that the type of postoperative consciousness disruption and the development of language disturbances may be related to the general anesthesia used during the procedure, damage caused to the brain tissue during electrode implantation, the placement of the electrodes, and the course of Parkinson’s [14,15]. However, the underlying molecular mechanism remains unexplored [16,17]. In order to present a new perspective on and new ideas for the clinical rehabilitation and treatment of patients after Parkinson’s surgery, we review the mechanisms of consciousness and speech impairment that occur after DBS from the perspective of neuroinflammation, offering solutions in the field of neuroprotection.

## 2. Activation of Astrocytes and Microglia Is the Beginning of Neuroinflammation

### 2.1. Activation of Microglia

Microglia serve as the brain’s primary immune effector cells and are involved in the immune response and nutrient provision of neurons [18]. Anesthetic and electrode locations can promote the production and release of pro-inflammatory factors in the peripheral immune system after DBS surgery in patients with Parkinson’s disease [19], which induces neuroinflammation [20].

Microglia can be activated into the M1 type through deposition [21]. Aβ is deposited in the hippocampuses of Parkinson’s patients, affecting their level of consciousness and memory [22]. Iron accumulates in the hippocampus because iron homeostasis is affected by the implantation of brain pacemakers. McCarthy’s study showed, that under neuroinflammatory conditions, microglia in the hippocampus can store intracellular iron released by heme catabolism and extracellular iron taken up by DMT1 [23,24]. Cells preferentially take up NTBI and IREs in response to proinflammatory stimuli, such as lipopolysaccharide (LPS) or-amyloid (Aβ) [23]. Iron-responsive elements (IREs) dissociate from their receptor-binding sites in the presence of high levels of iron [25], which prevents Aβ from catabolizing and causes it to aggregate. Consequently, the amount of Aβ in the hippocampus is further increased, activating microglia [26]. As DBS surgery requires the implantation of foreign bodies in the brain, this process destroys the blood–brain barrier, leading to lymphocyte activation, the production of reactive oxygen species, and the activation of microglia [27]. Additionally, anesthesia during DBS surgery may cause the nuclear factor B (NF-κ B) and calcineurin (Ca N) signaling pathways to assault the mitochondria and break the respiratory chain [28]. Since mitochondria are particularly susceptible to mitochondrial ROS damage, this triggers a vicious cycle [28]. This indicates that postoperative speech and awareness impairments are likely to occur after anesthesia [29]. Following surgery, the expression of miRNA-124 [30], which controls the majority of microglial functions, decreases, and the upstream vesicle-associated membrane protein 3 (VMAP3), whose expression is increased, significantly activates microglia and increases the release of associated inflammatory factors. Together, these factors influence the microglia, causing an imbalance in the activation of M1 and M2 phenotypes, which ultimately results in neuroinflammation [31]. Studies have shown that the level of sTREM2 in the cerebrospinal fluid (CSF) is correlated with the degree of microglial activation, suggesting that sTREM2 levels can be used to judge microglial activation [32].

### 2.2. Activation of Astrocytes

Mammalian brain astrocytes are among the most numerous brain cells, accounting for 20–40% of all brain cells [33]. Astrocytes may be activated during the perioperative phase via anesthesia and trauma response. Astrocyte activation occurs on both sides. Whether the effect is beneficial depends on the timing, microenvironment, and stimulation. Recent studies have shown that astrocytes play an important role in the maintenance of brain homeostasis. Astrocytes regulate the cerebral blood flow and synaptic electrical activity, which are also involved in supplying nutrients to neurons [34]. The materials used for the electrodes during DBS may cause immune cells to proliferate [35,36]. Electrodes used during DBS can also cause surrounding edema and activate astrocytes [37]. For example, the ability of the hippocampus to function normally may be affected by trauma-induced increases in glial fibrillary acidic protein (GFAP)-positive astrocytes [38]. Trauma during the perioperative period after DBS can activate astrocytes. Damage-associated molecular patterns (DAMPs) are released from cells, causing toll-like receptors (TLR) to regulate downstream signals. TLR3 is the major TLR expressed in human astrocytes, and its level increases in the presence of inflammatory stimuli [39]. TLR3 expressed on astrocytes and its ligands activates TLR3 downstream signaling, causing the induction and release of proinflammatory molecules, including cytokines, chemokines (CCL2, CCL3, CCL5, etc.) [40], and nitric oxide (NO), which destroy the blood–brain barrier (BBB) and activate astrocytes [41].

### 2.3. Crosstalk between Microglia and Astrocytes

**Figure 1:**
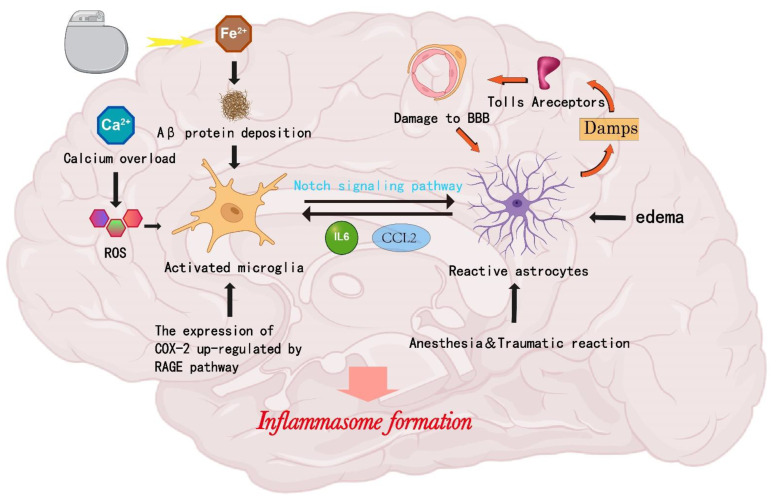
Activation and interaction of microglia and astrocytes. Neuroinflammation is initiated by the activation of microglia and astrocytes. Aβ is deposited in the brain as a result of the placement of electrodes during a DBS procedure, altering iron homeostasis and causing microglia to become activated. Due to an overabundance of calcium, ROS levels may also increase as a result of anesthesia during surgery. Additionally, anesthesia can boost COX-2 synthesis via the RAGE pathway, both of which have the capacity to activate microglia. Through the Notch pathway, activated microglia can boost astrocyte reactivity. The traumatic response after DBS surgery can induce astrocytes to generate DAMPs, which can then activate other astrocytes and trigger downstream signals controlled by TOLL receptors that may eventually result in BBB destruction. Edema brought on by DBS electrodes and anesthesia during surgery also activates astrocytes. By activating microglia through CCL2 and promoting microglia activation by secreting IL-6 and chemokines, activated astrocytes can also interact with microglia.

Both types of glial cells interact with each other by secreting large amounts of chemokines, cytokines, adenosine triphosphate, reactive oxygen species, and pro-inflammatory mediators to trigger a cascade response that contributes to inflammation [42]. Microglia and astrocytes can be activated into two phenotypes: pro-inflammatory (M1/A1) and anti-inflammatory (M2/A2) [42]. Several studies have shown that microglia can be activated by TLR4/Myd88/TRAF6/NF-κ B while upregulating the expression of Notch1 and its ligand Jagged1, causing an increase in astrocyte responsiveness [43]. Lambertsen’s experiments show that TNF- α is highly correlated with the crosstalk of microglia and astrocytes in the human brain. After being activated by trauma, microglia release TNF-α through the TLR4 pathway, directly triggering the astrocyte response by up-regulating their expression of TNF-α. There is also evidence to suggest that astrocytes fail to respond to pathogens or trauma without microglia [44,45]. Inflammatory factors released by microglial cells have an amplifying effect. These inflammatory agents (TNF-α, IL-1) amplify inflammatory activation through the special physiological structure of astrocytes [46]. Pascual et al. demonstrated that microglia rapidly release small amounts of ATP, and astrocytes, in turn, amplify this release, thereby increasing the frequency of excitatory postsynaptic currents through the ATP receptor P2Y1 [47]. Astrocytes may also activate microglia through CCL2 and increase the expression of CCR2 generated by microglia [48]. Astrocytes often interact with both neurons and blood vessels to maintain neuronal function and the blood–brain barrier. Astrocytes are major sources of chemokines including CCL2, CXCL1, CXCL10, and CXCL12 [49]. Microglial cells also express chemokine receptors, such as CCL2 and CXCL12 [50]. There is a strong association between microglia and astrocytes [48]. Other research has found that astrocytes phosphorylate protein kinase R–liker kinase (PERK) via action on Janus kinase 1 (JAK1), which promotes the downstream expression of thioredoxin-interacting protein (TXNIP) to secrete IL-6 and chemokines for microglial activation via the paracrine pathway [51]. In general, microglia are generally sensitive to trauma. First, M1-type microglia are activated. Furthermore, A1-type astrocytes serve as reserve forces that amplify neuroinflammation. TNF-α, IL-1β, C1a, and other inflammatory factors played a significant role. However, cellular communication between M2-type astrocytes and A2-type microglia plays an important role in the later stages of nerve repair (Figure 1).

## 3. Neuroinflammation Occurs as the Result of Multifactorial Synergy including Astrocytes and Microglia

### 3.1. Astrocytes Interact with Microglia to Induce Neuroinflammation

Microglia and astrocytes are important brain cells that maintain homeostasis and trigger immune responses. After activation, they affect the distribution and number of downstream immune factors, either individually or interactively, resulting in the formation of inflammasomes during neuroinflammation [52]. Microglia regulate the downstream myeloid differentiation factor-88 (Myd-88) and IL-1/TLR superfamily pathways through TNF-α to induce the release of IL-1, IL-6, IL-1β [53], and TNF-α, which is regulated by Glycogen synthase kinase3 (GSK-3) via the NF-α B pathway [54]. Microglia can be activated by peripheral inflammation, thereby producing excess reactive oxygen and reactive nitrogen [55] and activating downstream NOD-like receptor thermal-protein-domain-associated protein 3 (NLRP3) through the JNK1-mediated dephosphorylation of S194 [56]. Astrocytes play an important role in the activation of inflammation [57]. Their proliferation after trauma impairs neuroglial metabolic coupling and the release of S100β, leading to neuronal dysfunction [58]. In addition, astrocytes activated by microglia via TNF-α can also promote inflammation by releasing IL-17, NLRP3, apoptosis-associated speck-like protein containing a CARD (ASC), and other factors [59].

### 3.2. Other Factors Acting on Neurons May Induce Downstream Inflammation

In the nervous system, the blood–brain barrier plays an important role in protecting neurons from external factors and transmitting brain electrical signals smoothly [60]. After DBS surgery, problems in the DBS hardware may develop [35]; for example, the blood–brain barrier may be damaged because of DBS hardware problems [36], prompting the release of high levels of prostaglandin E2, oxygen radicals, and lysosomal enzymes that further amplify neuronal damage, affecting neurotransmitter production and promoting inflammation [61]. Owing to the dual effect of Parkinson’s disease and surgical trauma [21], Aβ accumulation can activate NLRP3 and upregulate the expression levels of IL-1β, IL-18, and Caspase-1 to cause inflammation [55]. By injuring the cell membranes of neuronal cell bodies and causing a potassium ion imbalance, NLRP1 is effectively activated to participate in the inflammatory cell death pathway [62]. Oxidative stress is an important surgical risk factor. Inhalation anesthesia isoflurane modulates both the CAN and NF- κ B pathways [28], attacking mitochondria to destroy the respiratory chain, promoting the accumulation of ROS in the process of oxidative stress, and subsequently activating the NLRP3 signal transduction pathway, thereby activating caspase-1 to participate in the inflammatory process [63].

## 4. Neuroinflammation-Induced Pyroptosomal Formation May Promote Consciousness Disorders

### 4.1. Inflammasome Formation

Pyroptosis is an important mechanism of programmed cell death, which belongs to the category of inflammatory cell death. It was discovered in 1992 and was officially named pyroptosis in 2001 [64]. This mechansim is often characterized by cell swelling, cell membrane rupture, and DNA damage [65]. Inflammasome formation is a hallmark of neuroinflammation and is the initial phase of pyroptosis [66,67]. As a multiprotein complex composed of pattern recognition receptors (PRRs), inflammasomes become activated following the recognition of DAMPs [68]. NLRP1, NLRP3, and NLRC4 play major roles in neuronal apoptosis in Parkinson’s disease [69]. Aβ can directly activate NLRP3 at the same time [55], causing intra-neuronal hypokalemia and triggering NLRP1 activation [62]. Cascaded ROS can specifically activate NLRP1 and subsequently activate caspase-1 [70]. At the same time, the activation of NLRP3 in microglia caused by anesthesia is also a key event [56]. Astrocytes may also promote inflammation via IL-17, NLRP3, ASC, and other factors [59]. NLRC4 further promotes the secretion of IL-1β and IL-18 from ASCs produced by astrocyte activation [71]. In addition, calcium overload within nerve cells due to neuroinflammation is one of the reasons for NLRP3 activation [72].

### 4.2. Pyroptosis Leads to Language and Consciousness Disorders

**Figure 2:**
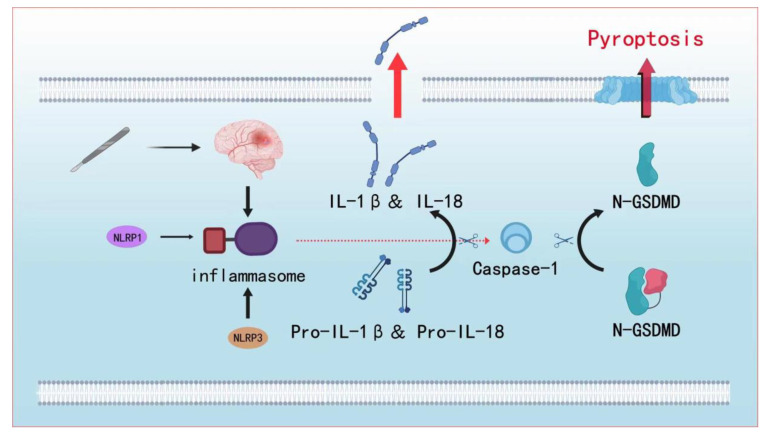
Inflammasome formation and the classical pyroptosis pathway. The triggering step of pyroptosis is the development of an inflammasome. Parkinson’s-disease-related inflammasome assembly is primarily regulated by NLRP1 and NLRP3. Inflammasomes may be formed when both of these factors come together with ASC. Pro-IL-1 and pro-IL-18 may further be broken down into IL-1 and IL-18 by activating caspase-1. In addition, caspase-1 has the ability to cleave GSDMD, separating its N- and C-termini. The N-GSDMD generated from this cleavage has the ability to generate holes in cell membranes, which makes it easier for IL-1 and IL-18 to be released, triggering a series of immunological reactions.

In the canonical inflammasome pathway, formed inflammasomes [73] bind to pro-caspase-1, pro-IL-1β, and pro-IL-18. Caspase-1 is activated first, followed by shearing to produce IL-1β and IL-18 [74]. IL-1β and IL-18 simultaneously cut GSDMD to separate its N- and C-termini. At this point, N-GSDMD is combined with membrane oligomerization, forming pores on the membrane [52], promoting the release of IL-1β and IL-18, and inducing a cascading immune response [75]. On the one hand, pyroptosis can affect brain functions, such as consciousness and language, through programmed neuronal death [76]. However, it also affects neuronal function and causes synaptic loss through the production of ROS, IL-1β, and IL-18 [77]. In addition to affecting the activation and distribution of microglia, astrocytes indirectly affect the internal environment of the nervous system and neurotransmitter production [78]. The hippocampus is an important organ for processing memory and consciousness in humans, and is more susceptible to damage from external sources because of its specific structure. Specifically, unlike other brain structures, the basal membrane covers only 30% of the vascular surface in the hippocampus, which facilitates the penetration of hormones and inflammatory factors into hippocampal neurons [79]. The use of ulinastatin to inhibit the release of pyroptosis-related molecules such as IL-1β and the inhibition inflammasome formation can effectively reduce the degree of inflammation in nerve cells [58]. In addition, cortical neurons, which are responsible for higher levels of consciousness and language, are also affected by pyroptosis [80]. Furthermore, its upstream target molecule, TNF-α, modulates the glutamate metabolism to produce excess glutamate, leading to toxicity, disturbing the balance between excitation and inhibition, and reducing neuronal excitability and synaptic activity [81] (Figure 2).

## 5. Therapeutic Prospects of Neuroprotection: Targeting the Microglia–Astrocytes Interaction to Regulate Neuroinflammation

Neuroprotection can delay disease progression by inhibiting inflammatory pathways, blocking the activation of astrocytes and microglia, reducing oxidative stress, and reducing neuronal apoptosis and pyroptosis [82]. Herein, we summarize the drugs and compounds that may exert such neuroprotective effects. Drugs with neuroprotective properties may reduce the development of neurological impairments after DBS and improve quality of life. In addition, significant amounts of peroxide are produced during DBS, resulting in peroxide accumulation (Table 1).

### 5.1. MAO-B Inhibitors

Monoamine oxidase (MAO) is a molecule produced in the brain whose oxidation product is hydrogen peroxide [83], which can be reduced by GSH in the brain. PD patients are known to have low GSH levels [84]. Furthermore, a significant amount of peroxide is produced during the DBS procedure, resulting in the accumulation of peroxide [28]. The accumulation of hydrogen peroxide generates hydroxyl radicals, depletes cellular antioxidants, and damages lipids, proteins, and DNA. Selegiline reduces the accumulation of oxidative stress by hydrogen peroxide, which enhances the action of superoxide dismutase (SOD) and catalase with endogenous antioxidant capacities, and further blocks the uptake of neurotoxins in nerve endings [85,86]. Rasagiline is a more potent MAO-B inhibitor than selegiline; it increases antioxidant capacity (SOD, catalase, and BCL-2) and reduces the toxic effects of peroxynitrite, MPTP, and 6-OHDA [87]. Rascol et al. previously showed that rasagiline improved the symptoms of patients [88].

### 5.2. Drugs That Inhibit the Activation of Microglia and Astrocytes

Cannabidiol (CBD) is one of more than 100 phytocannabinoids found in cannabis plants, accounting for 40% of the plant’s extract. It is not psychoactive, allowing it to exert anti-inflammatory effects while avoiding other adverse effects [89]. Cannabidiol exerts anti-inflammatory and neuroprotective effects by activating the TRPV1-CNTF pathway in astrocytes [90].

Dexmedetomidine (DEX) is used clinically as an α2-adrenoceptor agonist, as well as as a sedative [91]. Degos found that DEX can upregulate BDNF expression by relying on extracellular signal-regulated kinase (ERK), thereby reducing neuronal death [92]. Furthermore, DEX can inhibit activated microglia and astrocytes and reduce their secretion of inflammatory factors such as TNF- α and IL-1β [93], thereby exerting a neuroprotective effect.

Glucagon-like peptide-1 receptor (GLP-1R) agonists may potentially exert neuroprotective effects against nervous system diseases such as Alzheimer’s disease (AD) and Parkinson’s disease (PD). Previous studies have researched the intrastriatal injection of these agonists in sporadic PD. In α-syn PFF mouse models, the GLP-1R agonist NLY01 can block microglia activation and A1 astrocyte production [94]. Teramoto found that the GLP-1R agonist exendin-4 could prevent the activation of microglia both in vitro and in vivo [95].

Baicalein is a flavonoid that is mainly isolated from the roots of Scutellaria baicalensis, a plant belonging to the Labiatae family [96]. Previous studies have shown that baicalein attenuates astrocyte and inflammasome activation in a mitochondrial membrane permeability transition pore (MPTP)-induced PD model [97,98], and further inhibits the activation of the NF-κB signaling pathway and MAPK phosphorylation [99].

Androgens also exert neuroprotective effects [100]. The neuroprotective effects of dihydrotestosterone (DHT) occur vis the inhibition of inflammatory responses induced by LPS-activated microglia via the TLR4-mediated NF-κB and MAPK signaling pathways.

### 5.3. Neurotrophic-Factor-Based Drugs

In an MPTP-treated monkey model, glial-derived neuroadaptation factor (GDNF) contributed to the restoration of dopaminergic cell function [101]. MPTP was infused into the ventricles or striatum of MPTP-treated monkeys. The number and size of tyrosine hydroxylase (TH)-positive cells significantly increased [102]. This contributed to the improvement in brain cell damage during DBS.

Curcumin is a compound isolated from turmeric rhizomes that possesses anti-inflammatory [103] and antioxidant effects [104]. Curcumin stimulates the Trk/PI3K signaling pathway to restore neuronal regeneration and reduce the levels of tumor necrosis factor-α (TNF-α) and cystathionine activity, thereby increasing the levels of brain-derived neurotrophic factor (BDNF) and exerting neuroprotective effects [105].

### 5.4. Iron Chelator Drugs

Iron promotes the development of oxidative stress, leading to protein misfolding and Lewy body formation [23,106]. Iron chelators can collect and remove intracellular iron, thereby reducing ROS production and the misfolding and aggregation of certain proteins, such as α-synuclein. One study found that deferoxamine (DFO) and deferiprone (DFP) reduce iron levels in the brain and induce neuroprotection [107,108].

**Table 1:** Neuroprotective drugs that act by inhibiting neuroinflammation and via microglia–astrocytes interactions.

Targets	Drugs	Type of Drugs	Therapeutic Effect
MAO-B	Selegiline [87]	MAO-B inhibitor	Enhances antioxidant capacity and reduces oxidative stress
	Rasagiline [88]		
Microglia or astrocyte activation	Cannabidiol [89]	Plant cannabinoids	Activates the astrocyte TRPV1-CNTF pathway to exert anti-inflammatory and neuroprotective effects
	Dexmdetomdine [91]	α2-adrenoceptor	Upregulates the neurotrophin BDNF expression, and inhibits activated microglia and astrocytes
	NLY01 [95]	GLP-1R agonist	Blocks microglial activation and A1 astrocyte production
	Baicalein [96]	Flavonoid compound	Reduces astrocyte activation and inflammasome activation, inhibits the activation of NF-κB signaling pathway, and inhibits MAPK phosphorylation
	DHT [100]	Androgens	Microglia inhibit LPS activation causing inflammation induced by the TLR4-mediated NF-B and MAPK signaling pathway
GDNF	Curcumin [101]	Neurotrophic factor-based drugs	Stimulation of Trk/PI3K signaling to restore neuronal regeneration and reduce the levels of tumor necrosis factor-α (TNF-α) and caspase activity, thereby increasing the levels of brain-derived neurotrophic factor (BDNF)
Intracellular iron	DFO [109]	Iron chelator drugs	Removal of intracellular iron, thereby reducing ROS production and reducing misfolding and aggregation of certain proteins
	DFP [110]		

## 6. Hardware and Anesthesia of DBS Surgery

The selection of hardware and materials for DBS surgery is related to treatment outcomes. Previous research suggested that bleeding during DBS is caused by direct vascular damage during MER electrode propulsion [20]. Hardware-related infections also need to be addressed, as these occur in approximately 16% of cases; however, notably, the combination of a hydrogen dioxide solution and the IPG standby algorithm Isa seems to be an effective strategy to prevent IPG infections [111].

Regarding the selection of anesthesia for DBS surgery, it has been reported that general anesthesia can reduce the availability of electrophysiological activity and, in some cases, alter the characteristics of microelectrode recordings [112]. This prevents the identification of target structures [113]. However, recent clinical trials have shown that the sleep microelectrode-guided bilateral subthalamic nucleus DBS approach has similar outcomes to surgery on patients who are awake. Further, the incidence of cognitive, mood, and behavioral effects after surgery under local anesthesia was not higher than that after general anesthesia in this cohort [114]. In a meta-analysis comparing the efficacy of DBS under general and local anesthesia, the improvement in motor function of DBS under general anesthesia was comparable to that in the local anesthesia group [115].

## 7. Discussion

As a serious neurodegenerative disease [116], Parkinson’s disease is generally treated with drugs and surgery [117]. DBS has gradually become the first-line surgical treatment for Parkinson’s owing to its high patient tolerance and wide indications [118]. Among these, STN DBS is widely applicable, whereas GPi-DBS is the first choice when patients are relatively young and present only with symptoms during heavy exercise [119].

Although the surgical treatment of Parkinson’s is developing continuously, the level of patient satisfaction is not high, and only 43% of patients are completely satisfied with the results [12]. At one-year follow-up, more than half of the patients expressed negative emotions regarding the results [120]. The reason for this is that, after DBS surgery, patients experience significant damage to high-level nerve functions, including language disorders [14,121,122], and consciousness disorders [123,124]. This article focuses on the sequelae that appear after surgery, combined with the characteristics of the DBS surgical approach, material selection, anesthesia, and the research hotspots of cytokinesis POCD [125]. This study innovatively proposes that these sequelae may be related to the patient’s neuropathological changes that occur during Parkinson’s disease, the intraoperative approach, hardware, and the activation and interaction of microglia and astrocytes caused by anesthesia. Furthermore, we discuss how the inflammatory cytokines secreted by microglia and astrocytes lead to neuroinflammation by affecting the activation of the NF-κ B pathway, the release of inflammatory factors, and NLRP3/NLRP1 assembly through the classical or non-classical pyroptosis pathways. While this mechanism directly kills neurons and affects neurological function, it also causes a massive release of inflammatory factors through a cascade reaction that affects synaptic and neurotransmitter production.

We further explored the field of neuroprotection in relation to neuroinflammation, thereby improving the neurological functions that are impaired after DBS. In addition to traditional drugs, such as cannabidiol, which inhibit microglia and astrocyte activation, monoamine oxidase inhibitors affect oxidative stress and neurotrophic factors that act on dopaminergic neurons. The central mechanism of action of these drugs is to block the activation of factors during the pyroptosis of neuronal cells.

The role of ferroptosis in neurological impairment following DBS also requires further investigation. In clinical practice, novel iron chelators that reduce iron deposition during DBS surgery, such as nanocoated deferramine and curcumin, will be of great interest in future studies. Furthermore, α-Synuclein immunotherapy inhibits Lewy body production and improves neurological function. NPT200-11 from Neuropore Therapies and enterin kenterin are currently undergoing phase-two clinical trials. These drugs also have a relatively high potential to ameliorate potential damage. In addition to pharmacological methods, improving access to DBS surgery, using neuroendoscopy, etc., to reduce damage, changing the material composition of the brain pacemaker, and reducing the overall anesthesia time for DBS surgery will also benefit patients. Alternatively, there are several techniques that improve the efficiency and reduce the side effects of treatment, such as automated closed-loop adaptive DBS and multi-source stimulation. All of these are promising solutions. Percutaneous cerebrovascular intervention for neuromodulation is one of the most promising treatments for the future. Blood vessels are natural pathways in the human body. Although directional stimulation through the intravascular implantation of stents is less accurate, it causes less damage to the brain tissue, and the proportion of postoperative infection and consciousness disorders is also lower. Combining these techniques with AI technology may also be a possibility for the future development of DBS technology. Further, doctors can use artificial intelligence to accurately simulate and personalize DBS stimulation sites, so as to achieve more accurate neuromodulation. However, the use of AI assistance for positioning and manipulation during surgery can also reduce the possibility of consciousness disorders caused by postoperative brain injuries. The application of robotic surgery is of interest in the field of functional neurosurgery. In DBS surgery, the surgical robot can accurately locate the target before surgery and design its approach through artificial intelligence to accurately avoid important functional areas and blood vessels. At present, the ROSA surgical robot can improve the accuracy of the implanted electrode stimulator to within 0.4 mm and shorten the operation time, thereby greatly reducing the postoperative disturbance of consciousness caused by trauma.

## 8. Conclusions

DBS is an efficient and safe treatment that is widely used in functional neurosurgery and psychiatry. Herein, we reviewed the advantages of DBS over drug therapy and investigated the relationship between disturbance of consciousness, neuroinflammation, and pyroptosis after DBS. In addition, we reviewed the reliable pharmacological interventions for the relevant targets to alleviate disturbances in consciousness after DBS. Future developments in DBS surgery are anticipated.

## Data Availability

Not applicable.
